# NOSA, an Analytical Toolbox for Multicellular Optical Electrophysiology

**DOI:** 10.3389/fnins.2020.00712

**Published:** 2020-07-14

**Authors:** Sebastian Oltmanns, Frauke Sophie Abben, Anatoli Ender, Sophie Aimon, Richard Kovacs, Stephan J. Sigrist, Douglas A. Storace, Jörg R. P. Geiger, Davide Raccuglia

**Affiliations:** ^1^Institute of Neurophysiology, Charité – Universitätsmedizin Berlin, Corporate Member of Freie Universität Berlin and Humboldt-Universität zu Berlin, and Berlin Institute of Health, Berlin, Germany; ^2^German Center for Neurodegenerative Diseases, Charité – Universitätsmedizin Berlin, Berlin, Germany; ^3^School of Life Sciences, Technical University of Munich, Freising, Germany; ^4^Institute of Biology/Genetics, Freie Universität Berlin, Berlin, Germany; ^5^NeuroCure, Charité – Universitätsmedizin Berlin, Corporate Member of Freie Universität Berlin, Humboldt-Universität zu Berlin, and Berlin Institute of Health, Berlin, Germany; ^6^Department of Biological Science, Florida State University, Tallahassee, FL, United States

**Keywords:** voltage imaging, GEVI, analytical toolbox, multicellular activity, optical electrophysiology

## Abstract

Understanding how neural networks generate activity patterns and communicate with each other requires monitoring the electrical activity from many neurons simultaneously. Perfectly suited tools for addressing this challenge are genetically encoded voltage indicators (GEVIs) because they can be targeted to specific cell types and optically report the electrical activity of individual, or populations of neurons. However, analyzing and interpreting the data from voltage imaging experiments is challenging because high recording speeds and properties of current GEVIs yield only low signal-to-noise ratios, making it necessary to apply specific analytical tools. Here, we present NOSA (Neuro-Optical Signal Analysis), a novel open source software designed for analyzing voltage imaging data and identifying temporal interactions between electrical activity patterns of different origin. In this work, we explain the challenges that arise during voltage imaging experiments and provide hands-on analytical solutions. We demonstrate how NOSA’s baseline fitting, filtering algorithms and movement correction can compensate for shifts in baseline fluorescence and extract electrical patterns from low signal-to-noise recordings. NOSA allows to efficiently identify oscillatory frequencies in electrical patterns, quantify neuronal response parameters and moreover provides an option for analyzing simultaneously recorded optical and electrical data derived from patch-clamp or other electrode-based recordings. To identify temporal relations between electrical activity patterns we implemented different options to perform cross correlation analysis, demonstrating their utility during voltage imaging in *Drosophila* and mice. All features combined, NOSA will facilitate the first steps into using GEVIs and help to realize their full potential for revealing cell-type specific connectivity and functional interactions.

## Introduction

One goal of The American BRAIN initiative was to develop methods to comprehend complex activity patterns in specific brain networks and even in whole brains (Alivisatos et al., 2012). One crucial step toward gaining insight into the mechanisms and interactions of neural activity patterns is the development of software that allows for measuring multi-cellular electrical activity and analyzing their complex datasets (Alivisatos et al., 2012).

Genetically encoded voltage indicators (GEVIs) have emerged as promising tools for measuring neural electrical activity (Lin and Schnitzer, 2016; Yang and St-Pierre, 2016). GEVIs are powerful, in part because they can be genetically targeted to specific neural populations and optically report the electrical activity from many neurons simultaneously, and even from neuropil that is otherwise inaccessible to classical electrophysiology. GEVIs have been successfully used for monitoring multicellular activity and population dynamics in *Drosophila* (Cao et al., 2013; Raccuglia et al., 2016, 2019; Aimon et al., 2019), visual and olfactory responses in mice (Gong et al., 2015; Storace et al., 2015; Storace and Cohen, 2017), cerebellar activity in Zebrafish (Miyazawa et al., 2018) and also pharyngeal activity in *C. elegans* (Azimi Hashemi et al., 2019).

Although GEVIs are being continually improved (Lin and Schnitzer, 2016; Storace et al., 2016), high recording speeds, low signal-to-noise ratios (SNR) and GEVI-specific kinetics bring about unique challenges with respect to data analysis (Yang and St-Pierre, 2016; Kulkarni and Miller, 2017) and thus require the development of adequate processing software. Yet, there is currently no freely available software, which combines processing tools addressing these challenges with analytical tools for identifying specific activity patterns, temporal relations and functional interactions.

Here we present NOSA (Neuro-Optical Signal Analysis) – an open source software designed specifically for the analysis of multicellular optical electrophysiology. NOSA features baseline fitting and filtering algorithms to extract electrical patterns from high speed recordings with low SNR. Moreover, NOSA provides analytical tools for identifying specific activity patterns and their temporal relation via functions that provide spectral and cross-correlation analysis. NOSA also includes features for spike- and burst detection, movement artifact compensation, and the ability to analyze simultaneously performed optical and electrical recordings. With these analytical tools, intuitive design, and convenient graphical interface, NOSA should greatly facilitate the first steps into using GEVIs, enabling laboratories around the world to perform and analyze multicellular voltage imaging recordings.

## Materials and Methods

### NOSA Software

NOSA was written in Python 3.7.1 (see https://docs.python.org/3/license.html for license information) and only runs on Windows. Besides default packages and built in functions, NOSA uses a variety of additional packages (Table 1). The executable NOSA file, the master code, a manual containing additional information on the graphical user interface, the function of specific features, performance aspects and workflow examples are provided under the following GitHub repository: https://github.com/DavideR2020/NOSA. To obtain example files please send an email to the lead contact.

**TABLE 1:** Packages used for NOSA.

Name	Version	License type	Copyright notice	Website
NumPy	1.16.2	BSD	Copyright© 2005-2018, NumPy Developers. All rights reserved.	https://www.numpy.org
SciPy	1.2.1	BSD	Copyright© 2001, 2002 Enthought, Inc. All rights reserved. Copyright© 2003-2013 SciPy Developers. All rights reserved.	https://www.scipy.org
PyQt5	5.11.3	GPL v3	Copyright© 2018 Riverbank Computing Limited	https://www.riverbankcomputing.com/software/pyqt/
PyQtGraph	0.10.0	MIT	Copyright (c) 2012 University of North Carolina at Chapel Hill Luke Campagnola (luke.campagnola@gmail.com)	http://www.pyqtgraph.org
Matplotlib	3.0.3	PSF-based	©Copyright 2002 - 2012 John Hunter, Darren Dale, Eric Firing, Michael Droettboom and the Matplotlib development team; 2012 - 2018 The Matplotlib development team	http://matplotlib.org
pyStackReg	0.2.1	Custom	C + + Port of the TurboReg ImageJ Plugin. Original code by Philippe Thevenaz (Thevenaz et al., 1998). Porting by Gregor Lichtner	https://bitbucket.org/glichtner/pystackreg
DIPY (Garyfallidis et al., 2014)	0.15.0	Revised BSD-2-Clause	Copyright (c) 2008-2019, dipy developers. All rights reserved.	http://dipy.org
QDarkStyle	2.5.4	MIT	Copyright (c) 2013-2018 Colin Duquesnoy.	https://github.com/ColinDuquesnoy/QDarkStyleSheet
Neo	0.7.1	BSD-3-Clause	Copyright (c) 2010-2018, Neo authors and contributors.	http://neuralensemble.org/neo
Quantities	0.12.3	BSD	Copyright (c) 2012, Darren Dale dsdale24@gmail.com All rights reserved.	http://python-quantities.readthedocs.io/
XlsxWriter	1.1.5	BSD	Copyright (c) 2013, John McNamara jmcnamara@cpan.org All rights reserved.	https://github.com/jmcnamara/XlsxWriter
Tifffile	2019.3.8	BSD	Copyright (c) 1994-2019, Christoph Gohlke. All rights reserved.	https://www.lfd.uci.edu/∼gohlke/
PyInstaller	3.4	GPL-based	Copyright (c) 2010-2019, PyInstaller Development Team Copyright (c) 2005-2009, Giovanni Bajo Based on previous work under copyright (c) 2002 McMillan Enterprises, Inc.	http://www.pyinstaller.org/
Markdown	1.0.1	BSD-style	Copyright© 2004, John Gruber http://daringfireball.net/ All rights reserved.	https://daringfireball.net/projects/markdown/
Markdown-styles	3.1.10	BSD-3-Clause	none provided – created by GitHub user mixu (Mikito Takada)	https://github.com/mixu/markdown-styles

### Optical and Classical Electrophysiology of R5 Neurons in *Drosophila melanogaster*

Flies (*Drosophila melanogaster*) were reared on standard cornmeal food at 25°C and 60% humidity under a 12 h light/dark regime. All flies were obtained from the Bloomington Stock resource center (BDRC). Experiments were performed with 3-10 d old female flies at ZT 8-12 (Zeitgeber time; during a 12/12 h light/dark cycle the onset of light is at ZT 0 h and the offset of light is at ZT 12 h). Whole-brain explant dissections and fly *in vivo* preparation were performed as previously described (Cao et al., 2013; Owald et al., 2015). During *ex vivo* experiments (Figures 3, 4) external solution consisted of (in mM): 90 NaCl, 3 KCl, 1.5 CaCl_2_, 5 MgCl_2_, 1 NaH_2_PO_4_, 10 glucose, 10 sucrose, 8 trehalose, 5 TES and 26 NaHCO_3_. During *in vivo* experiments (Figures 5, 6) external solution consisted of (in mM): 70 NaCl, 3 KCl, 1.5 CaCl_2_, 20 MgCl_2_, 1 NaH_2_PO_4_, 10 glucose, 10 sucrose, 8 trehalose, 5 TES, and 26 NaHCO_3_. For a fully functional Mg^2+^ plug, Drosophila NMDA receptors require 20 mM of Mg^2+^ (Miyashita et al., 2012). To substitute for missing sensory input in the *ex vivo* preparation, we reduced external Mg^2+^ to 5 mM which leads to spontaneous activity in R5 neurons (Raccuglia et al., 2019).

Imaging was performed on an Olympus BX51WI microscope using a Plan Apochromat 40×, numerical aperture 0.8, water-immersion objective (Olympus, Japan). ArcLight was excited at 470 nm using a Lumencor Spectra X-Light engine LED system. LED power was adjusted for each recording individually to make sure that fluorescent images were not saturated. The objective C-mount image was projected onto an Andor iXon-888 camera controlled by Andor Solis software. Imaging was performed at frame rates of 80 Hz (Figures 3A–C, 5, 6), 160 Hz (Figures 3D–G), 250 Hz and 1000 Hz (Figure 4).

*In vivo* (Figure 5) whole-cell patch-clamp recordings from R5 neurons were performed at ZT 8-12 as reported elsewhere (Wilson and Laurent, 2005; Murthy and Turner, 2013; Donlea et al., 2018). Neurons were recorded for up to 5 min. Identification of R5 neurons was based on ArcLight expression. External saline was used as described above. Patch pipettes (7–10 MΩ) were filled with internal saline containing (mM): 135 K-aspartate, 10 HEPES, 1 EGTA, 1 KCl, 4 MgATP, 0.5 Na3GTP. Internal solution was adjusted to a pH of 7.2, with an osmolarity of 265 mmol/kg.

### Ca^2+^ Imaging of Hippocampal Slices

Animal care and handling was in accordance with the Helsinki declaration and institutional guidelines. Protocols for organ removal and culturing were approved by the State Office of Health and Social Affairs Berlin, under the license number T0123/11. Organotypic hippocampal slice cultures were prepared as described previously (Kann et al., 2011; Prager et al., 2019). In short, hippocampal slices (400 μm) were obtained from Wistar rat pups at postnatal day 6–7. Slices were positioned on cell culture membrane inserts (Millicell-CM, Millipore) and maintained for 6 days in culture medium (50% MEM, 25% HBSS, 25% Horse Serum and 1 mM L-glutamine, pH set to 7.3) at 5% CO_2_. At day 7, slice cultures were bulk stained with OGB-1-AM BAPTA (5 μM in DMSO, 0.01% Pluronic F-127, 0.005% Cremophor) by submerging them for 50 min at room temperature in carbogen bubbled serum free medium.

Fluorescence recordings were obtained with a spinning disk confocal microscope (Andor Revolution, BFI Optilas GmbH, Gröbenzell, Germany), equipped with an EMCCD camera (Andor iXonEM+, 60x objective N.A.1, 2 x binning, 20 Hz recording speed, laser intensity 150–200 μW at the focal level). Slice cultures were superfused with carbogen (95% O_2_, 5% CO_2_) saturated artificial cerebrospinal fluid containing (in mM): 129 NaCl, 3 KCl, 1.25 NaH_2_PO_4_, 1.8 MgSO_4_, 1.6 CaCl_2_, 26 NaHCO_3_, and 10 glucose (pH 7.4, t = 30°C). For induction of synchronized epileptiform activity, Mg^2+^ was omitted and KCl was elevated to 5 mM (Prager et al., 2019).

### Whole Brain Voltage Imaging in Drosophila

Whole brain recordings were performed using light field microscopy as described in detail elsewhere (Aimon et al., 2019). In short, a modified upright Olympus BX51W with a 20x NA 1.0 XLUMPlanFL (Olympus) was used. An adequate microlens array (RPC Photonics) positioned at the image plane and two relay lenses (50 mm f/1.4 NIKKOR-S Auto from Nikon) projected the image onto the sensor of a scientific CMOS camera (Hamamatsu ORCA-Flash 4.0). A 490 nm LED (pE100 CoolLED) at approximately 10% of its full power was used for excitation. As filter set we used a 482/25 bandpass filter, a 495-nm dichroic beam splitter, and a 520/35 bandpass emission filter (BrightLine, Semrock). The recording was performed at a frame rate of 200 Hz. The whole brain volume was reconstruction from the light field image as described in Aimon et al. (2019).

### Voltage and Calcium Imaging in the Olfactory Bulb

Olfactory bulb recordings in mice were performed as described in detail elsewhere (Storace et al., 2015). In short, C57BL/6 mice (JAX, Bar Harbor, MA) were injected into the olfactory bulbs with AAV1 expressing either ArcLight- or GCaMP6f. Mice were anesthetized (ketamine/xylazine) and the bone above both olfactory bulbs was either thinned or removed. The exposure was covered with agarose and sealed with a glass coverslip. The dorsal surface of both hemispheres was illuminated with 485 ± 25 nm light using epifluorescence illumination on a Leitz Ortholux II microscope with a tungsten halogen lamp or a 150 W Xenon arc lamp (Opti Quip) and a 515 nm long-pass dichroic mirror. Fluorescence emission was recorded with a NeuroCCD-SM256 camera with 2 × 2 binning at 125 Hz using NeuroPlex software (RedShirtImaging, Decatur, GA). All surgical procedures were approved by the Yale IACUC.

## Results

### NOSA Interface and Overview of Processing Tools

**FIGURE 1:**
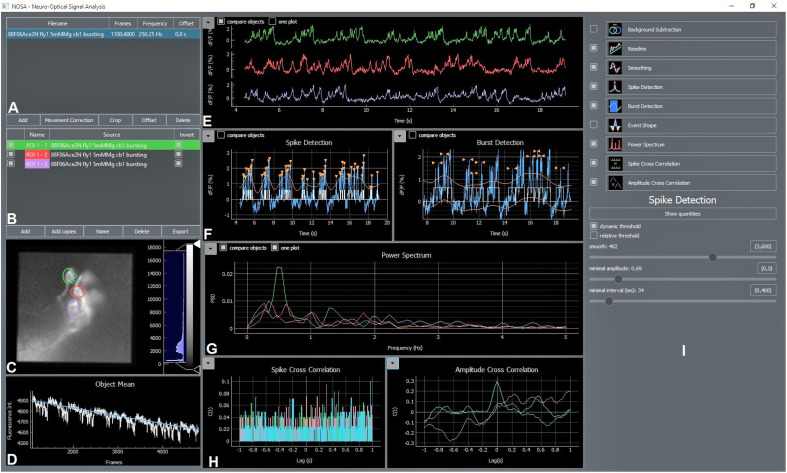
NOSA Interface. **(A)** The file manager shows imported files and contains options for movement correction, temporal cropping, offset correction and interpolation of the recording speed. **(B)** ROI manager showing added ROIs, which can be renamed and deleted. ROI masks can be generated by copying ROIs into other files. **(C)** Video panel showing currently selected frame and added ROIs. The recording can be adjusted for brightness and contrast. **(D)** Average fluorescence intensity of selected ROIs with baseline fitting curve and slide for selecting specific frames of the recording. **(E)** Processed data showing relative changes in fluorescence over time. Calculation of the relative change in fluorescence is based on recording speed, background subtraction, baseline correction and smoothing. **(F)** Panel for semi-automatic detection of spikes and bursts. Detected events are indicated with orange arrows and white lines/squares indicating spikes/bursts. **(G)** Power spectrum analysis based on processed data. If the event shape feature is selected this panel also shows the average shape of detected events. **(H)** Panel showing spike cross correlation calculated based on detected spikes and amplitude cross correlation calculated based on relative changes in fluorescence. **(I)** Control panel for adjusting the settings of each feature.

Our software package NOSA (Figure 1) is designed to process and analyze voltage imaging recordings. Recordings can be imported into NOSA as tif/tiff files, which can be temporally cropped (the user can select a specific time window) or corrected for movement artifacts (Figure 1A). NOSA automatically calculates the relative changes in fluorescence for selected regions of interest (ROIs) (Figures 1B,C) after factoring in recording speed, background correction as well as selected fitting (e.g., exponential drift correction) and filtering algorithms (Figure 1D). Activity patterns from different ROIs (e.g., cells) can be displayed in one plot to facilitate comparisons, although the user can easily switch to a more detailed view of the currently selected ROI (Figure 1E).

Because most GEVIs exhibit decreases in their fluorescence in response to depolarization, NOSA provides the option to invert the relative changes in fluorescence (Figure 1B). To generate ROI masks, selected ROIs can be copied and pasted into other recordings (Figure 1B). All data extracted by NOSA can be exported as spreadsheet files (Figure 1B). As high recording speeds generate a larger number of frames, we included simple but efficient options to increase software performance. For example, the recording frequency can be reduced by applying different interpolation algorithms (Figure 1B, right-click on file name, see next chapter for details). Moreover, software performance can be increased by deselecting the live preview which will suspend processing of changes while moving the ROI. Within each uploaded recording there is a default square ROI. Additional ROIs can be added by pressing the “add” button and the ROI shape can be changed with a right click (Figure 1B).

NOSA features power spectrum analysis as well as spike and burst detection to analyze activity patterns (Figures 1F,G). The event shape feature uses detected events to display the average firing characteristics of a neuron. To analyze temporal relations and functional interactions between activity patterns, cross correlation can be performed on detected events and on the relative changes of neural activity in optical and electrical recordings (Figure 1H). Via the control panel all features can be controlled (Figure 1I) for each ROI independently and settings selected for one ROI can easily be applied to all other ROIs.

### NOSA Workflow

**FIGURE 2:**
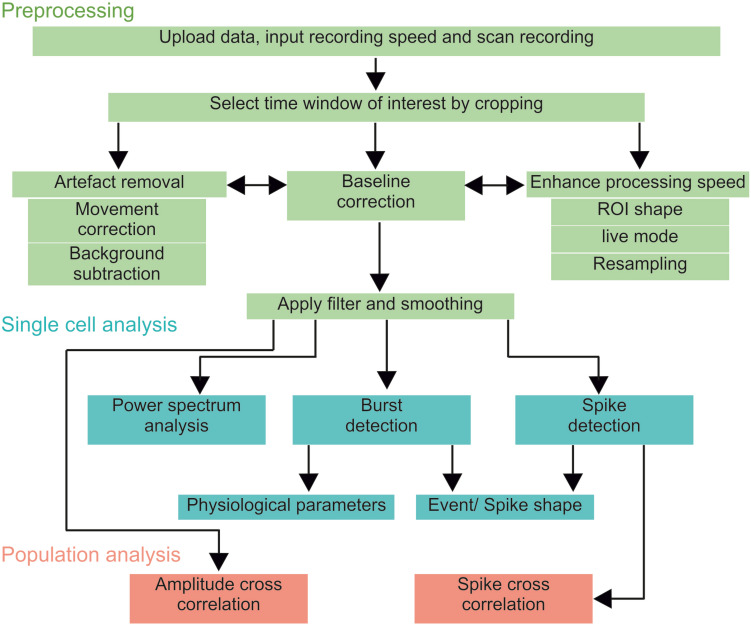
NOSA workflow. After uploading a recording, it should first be scanned for a time window of interest and potential artifacts induced by movement or light scattering. After adjusting the processing speed, cropping and artifact removal, the user should apply the baseline correction, filtering and smoothing. After these preprocessing steps, single cell physiological parameters can be analyzed via power spectrum analysis, burst and spike detection. To identify functional interactions between neurons (population analysis) the amplitude cross correlation uses the relative changes in fluorescence while the spike cross correlation uses the temporal relation between detected spikes.

NOSA provides an optimal workflow that facilitates the analysis of optical multicellular data (Figure 2), while also providing the flexibility to easily switch back and forth between different features. After uploading the data, the user should scan the recording for a time window of interest and apply the temporal cropping feature. If necessary, the processing speed can be enhanced by deactivating the live preview mode, by using a square ROI and by reducing the number of data points via resampling the recording frequency (adjust frequency, right click on file name). Resampling can also be used to unify different recording speeds, facilitating the simultaneous evaluation of multiple recordings. However, this method should only be used when the recording frequency is higher than needed to resolve the shape of single events or the interval between events. Therefore, NOSA will suggest a resampling frequency based on the temporal relation between detected events (spike detection) and their duration (burst detection). Based on the Nyquist theorem, the resampling frequency for resolving event intervals should be twice as high as the signal frequency. For resolving single events, the resampling frequency should not be below 10 times faster than the shortest event. At this point, light scattering artifacts can be corrected for by using the background correction function. If the light scattering artifact is due to a common source, then the ROI background subtraction should be applied. However, in multicellular recordings, light can scatter from multiple sources. In this case, the perisomatic background correction should be used. From here, movement related artifacts can be corrected by using a movement correction algorithm. The specific algorithm choice strongly depends on the type of movement. NOSA provides a side-by-side comparison of the original and corrected data for a convenient visual assessment of whether the correction was successful. For assessing how the movement correction affects the signal-to-noise ratio the corrected recording can be saved as a separate file and uploaded alongside the original file.

In the next step the user should correct for shifts in baseline fluorescence by applying the baseline correction (Figure 2). To help the user to pick the optimal algorithm and parameters, NOSA provides visual assistance by displaying the fitting curve next to the fluorescence intensity of the selected ROI (Figure 1D). Next, filtering and smoothing can be applied, although the specific choice of filter and parameter are highly dependent on the type of data and recording parameters (Widmann et al., 2015). The consequences of changing the filter and filter parameters are directly visualized in NOSA (Figure 1E), so that the desired frequency components can be isolated and noise can be smoothed without affecting the amplitude and shape of specific events. To facilitate a uniform analysis within and between recordings, the selected preprocessing algorithms and parameters are applied for each subsequently added ROI.

At this point, a power spectrum analysis and burst/spike detection can be applied (Figure 2), where static and dynamic thresholds (either set manually or based on the standard deviation of the noise and overall average) provide semi-automatic methods for detecting neural events. The optimal parameters for threshold selection depend on the variation of amplitudes and the signal-to-noise ratio and should therefore be determined empirically via visual assistance provided by NOSA (Figure 1F). Both burst and spike detection can be used to generate the average shape of all detected events. The burst detection can be used to quantify single cell physiological parameters such as burst duration, maximum amplitude (A_max_), time until peak (t_peak_) and the time constant of the decay from A_max_ (τ_decay_) (Chamberland et al., 2017). For τ_decay_ we used non-linear least squares to fit the exponential function f(x) = a^∗^e^–b^*x^ to the data after A_max_ and set τ_decay_ = 1/b. These parameters can be used to analyze depolarization phases as well as hyperpolarization phases. The spike detection also automatically quantifies spike amplitude and τ_decay_.

For population analysis, the spike cross correlation uses detected spikes to identify the temporal relation of activity patterns between neurons. In contrast, the amplitude cross correlation uses the relative changes in fluorescence to identify functional interactions and can therefore be performed after preprocessing. We have also implemented the option to perform a cross correlation on instantaneous amplitudes which employs the Hilbert transform of recorded activity and is particularly useful for identifying temporal relations in local field potential and compound recordings (Adhikari et al., 2010). Moreover, band pass filtering can be applied for comparing temporal relations within a specific frequency range.

### Multicellular Optical Electrophysiology

**FIGURE 3:**
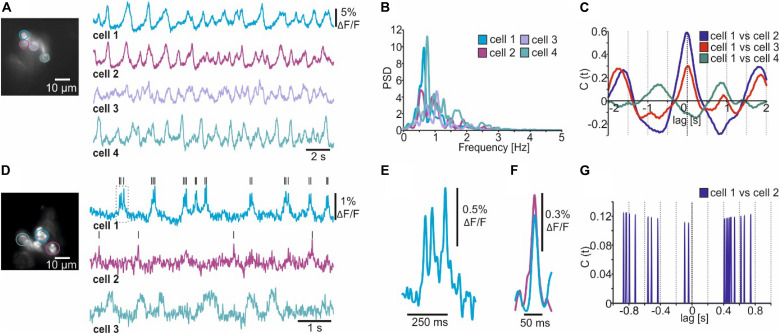
Analyzing multicellular electrical activity from optical recordings performed in *Drosophila melanogaster*. **(A)** Wide-field image and single-cell membrane potential oscillations of *Drosophila* R5 ring neurons expressing the GEVI ArcLight. **(B)** Power spectrum analysis of R5 electrical activity shown in A. **(C)** Amplitude cross correlation indicating the temporal relation of R5 electrical patterns shown in A. **(D)** Wide-field image and single-cell electrical activity of fan-shaped body neurons expressing the GEVI Varnam. Lines indicate detected spikes using a linear threshold approach. **(E)** Burst (see dashed square in D) showing that Varnam resolves single spikes during bursting. **(F)** The event shape feature shows the average kinetics of the action potentials detected in cell 1 and 2 [see black lines in **(D)**]. **(G)** Spike cross correlation indicating no temporal relations between spikes detected in **(D)**.

To demonstrate the utility of NOSA we used *Drosophila* to express the genetically encoded voltage indicator (GEVI) ArcLight in ellipsoid body R5 ring neurons and the GEVI Varnam in fan-shaped body neurons (Figure 3). Both neural structures are considered to be integration centers for various sensory modalities (Seelig and Jayaraman, 2013; Green et al., 2017; Sun et al., 2017; Hu et al., 2018) and play important roles in locomotion (Strauss and Heisenberg, 1993) and sleep regulation (Donlea et al., 2014, 2018; Liu et al., 2016; Guo et al., 2018; Raccuglia et al., 2019).

After setting a ROI, the optical trace should first be corrected for shifts in baseline fluorescence (e.g., due to photoisomerization and bleaching). Several baseline correction algorithms are included (Supplementary Figures S1–S3), and the user can set additional baseline markers to guide the fitting curve for more complex shifts in baseline fluorescence (Supplementary Figure S1A). After baseline correction, smoothing and inversion of the raw fluorescence (depolarization leads to a reduction in fluorescence), the electrical patterns of single R5 neurons become apparent (Figure 3A, compare Supplementary Figure S1). A power spectrum analysis revealed that single R5 neurons oscillate between 0.5 – 1.5 Hz (Figure 3B). We recently reported that R5 oscillations within this spectrum are linked to the fly’s sleep quality because they facilitate consolidated sleep phases (Raccuglia et al., 2019). The cross-correlation function built into NOSA provides a simple way of visualizing the temporal relation between the electrical patterns of the different cells (Figure 3C). The cross correlogram indicates that electrical patterns of cells 1, 2 and 3 largely overlap (main phase lag at 0) while cell 4 is out of phase (Figure 3C).

Due to the relatively low recording speed (78 Hz) and the slow kinetics of ArcLight we could not resolve single spikes in this example (Figure 3A). By increasing the recording speed to 160 Hz and taking advantage of the improved kinetics of the red-shifted GEVI Varnam (Kannan et al., 2018), we resolved single action potentials within the electrical activity of dorsal fan-shaped body neurons (Figures 3D,E). Compared to ArcLight, the SNR is lower and thus the detection of spikes heavily depends on the imaging conditions (Figure 3D), as individual spikes could not be resolved in the dimmer cells (Figure 3D, see cell 3).

That said, several functions are included to facilitate spike detection in low signal-to-noise recordings. This includes several filtering algorithms (see next chapter) as well as the ability to semi-automatically detect spikes and visualize their average shape using the event-shape feature (Figure 3F, compare Supplementary Figure S2). Moreover, the temporal relation between detected spikes can be analyzed using the spike cross correlation feature (Figure 3G, compare Supplementary Figure S2).

### Spike and Burst Detection

**FIGURE 4:**
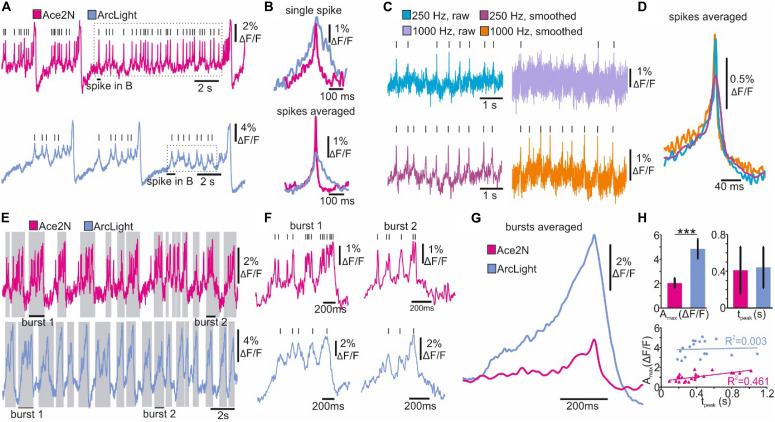
Spike and burst detection revealing kinetic properties of GEVIs and neuron-specific firing characteristics. **(A)** Optical recordings of spiking *Drosophila* R5 neurons expressing the GEVIs Ace2N and ArcLight. Lines indicate detected spikes using a linear threshold approach. **(B)** Single and averaged spikes selected from recordings in A (dashed square indicates spikes selected for event shape). The event shape feature indicates improved kinetics of Ace2N. **(C)** Optical recordings of a spiking *Drosophila* R5 neuron (Ace2N) performed at different recording speeds. Lines indicate identified spikes. **(D)** The event shape feature shows the average kinetics of spikes detected in C. **(E, F)** Optical recordings of bursting *Drosophila* R5 neurons expressing the GEVIs Ace2N and ArcLight. Gray shaded areas and lines indicate detected bursts and spikes using the dynamic threshold approach. **(G)** The event shape feature generates the averaged kinetics of detected bursts indicating GEVI-specific differences in the optical representation of bursts. **(H)** Quantified burst parameters show increased maximum response amplitude (A_max_) for ArcLight and a similar time to peak response (t_peak_). Regression analysis indicates a correlation between A_max_ and t_peak_ for Ace2N but not for ArcLight. Statistics: Students *t*-test, *n* = 16–20, *p* < 0.001.

In NOSA, electrical characteristics of single neurons as well as the kinetics of different GEVIs can be analyzed in detail using event detection features. To demonstrate this, we compare the GEVIs ArcLight and Ace2N (Gong et al., 2015) in R5 neurons in *Drosophila*. While most R5 neurons burst (∼90%), some mainly spike (Liu et al., 2016). NOSA’s spike detection and event shape feature was used to analyze two spiking R5 neurons expressing either ArcLight or Ace2N (Figures 4A,B). While the kinetics of the depolarization are comparable, the repolarization is considerably slower in ArcLight, which is in accordance with previous findings indicating that Ace2N has faster kinetics (Gong et al., 2015).

Optical representations of action potentials do not only depend on the GEVI kinetics, but also on a sensitive interplay between recording-speed and signal-to-noise ratio. To demonstrate this, action potentials were imaged using Ace2N at either 250 Hz or 1000 Hz (Figure 4C). The faster recording speed resulted in a drastically reduced signal-to-noise ratio, making action potentials barely detectable (Figure 4C). However, filtering algorithms provided in NOSA can increase the signal-to-noise ratio and thus the spike detection fidelity, as demonstrated here by using the Savitzky-Golay algorithm (Figure 4C). Automatic averaging of detected spikes (event shape) indicates that the temporal features of single action potentials are well represented at 250 Hz (Figure 4D). Here, the limiting factors seem to be the temporal dynamics of the GEVI itself.

An advantage of enhanced temporal dynamics of a GEVI becomes apparent when analyzing high-frequency spikes in bursting neurons (Figure 4E). Using Ace2N, spikes riding on top of bursts are more likely to be resolved and are therefore more readily detectable (Figure 4F). This is likely due to the slower kinetics of ArcLight, resulting in several spikes probably merging into one “spike”. However, the event shape feature indicates that the temporal characteristics of the bursts are identical, but ArcLight produces a bigger change in relative fluorescence (Figure 4G).

The burst detection feature can be used to directly quantify various response parameters of bursts or other detected events (Figure 4H). These parameters demonstrate that the maximum response amplitude (A_max_) in ArcLight expressing neurons is significantly increased while the time to peak response (t_peak_) is similar. Performing a regression analysis on these parameters indicates that in Ace2N A_max_ increases with t_peak_ while there is no such correlation in ArcLight (Figure 4H).

### Movement and Background Correction

**FIGURE 5:**
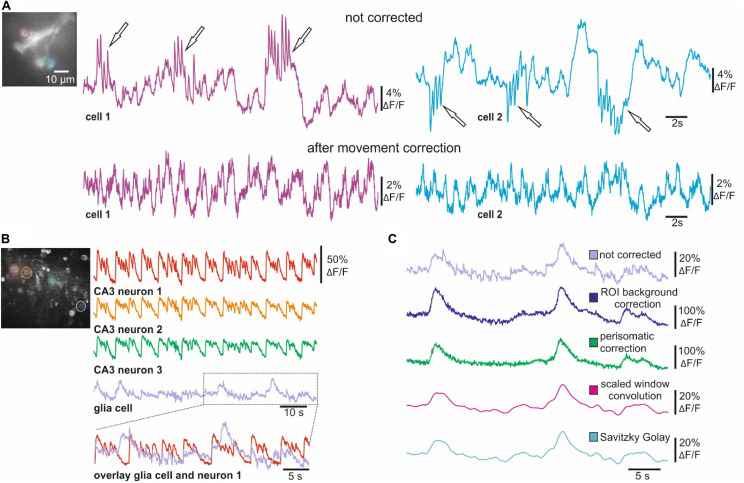
Movement and background correction for voltage and Ca^2+^ imaging. **(A)** *In vivo* voltage imaging (ArcLight) of single *Drosophila* R5 neurons before and after performing the symmetric diffeomorphic movement correction. **(B)** Ca^2+^ imaging of CA3 neurons and glia cells from hippocampal slices of Wistar rats. CA3 neurons indicated in the wide-field image show synchronous activity of CA3 neurons after inducing epileptiform activity. The overlay demonstrates how CA3 activity contaminates the optical representation of glial activity. **(C)** Activity of glia cell indicated in B before and after ROI background correction, perisomatic background correction and smoothing with scaled window convolution as well as with the Savitzky Golay algorithm.

Due to a relatively small SNR, movement artifacts are especially problematic for *in vivo* voltage imaging. We therefore implemented several movement correction algorithms into NOSA. We here demonstrate the symmetric diffeomorphic algorithm, which was originally designed for detecting brain deformations during magnetic resonance imaging (Avants et al., 2008). This algorithm maximizes the cross correlation within the space of diffeomorphic maps by using inverse transformations and the Euler-Lagrange equation (Avants et al., 2008). This sophisticated algorithm is very time consuming but shows impressive results (Figure 5A). Single ArcLight expressing R5 neurons recorded *in vivo* in *Drosophila* show substantial movement artifacts (Figure 5A). However, after performing the symmetric diffeomorphic algorithm electrical activity can be recovered even during periods of heavy movement.

Scattering light can also reduce the signal-to-noise ratio in epifluorescence imaging experiments. This is especially problematic in bulk dye-loading procedures as Ca^2+^- or voltage sensitive dyes accumulate differently in different cell-types (i.e., neurons and glial cells), leading to a large variability in fluorescence intensity. To address this issue, we implemented ROI and perisomatic background correction algorithms into NOSA (Figures 5B,C). To demonstrate their utility, we analyzed epileptiform activity in hippocampal slices of Wistar rats loaded with the Ca^2+^ indicator OGB-1-AM (Kovacs et al., 2001; Figure 5B). Here, strong and synchronized increases in fluorescence in CA3 neurons lead to increased light-scattering, contaminating the optical representation in a simultaneously recorded glial cell (Figure 5B). However, ROI and perisomatic background correction both successfully reduce the effects of scattered light from surrounding neurons, increasing the SNR in the activity pattern of a glial cell (Figure 5C). In comparison, smoothing algorithms can also eliminate the higher frequency components generated by scattering light but fail to eliminate the slower components and distort the glial Ca^2+^ responses (Figure 5C). Therefore, the user should first employ the background correction and then use moderate smoothing to remove residual noise (Figure 2).

### Combined *in vivo* Optical and Classical Electrophysiology

**FIGURE 6:**
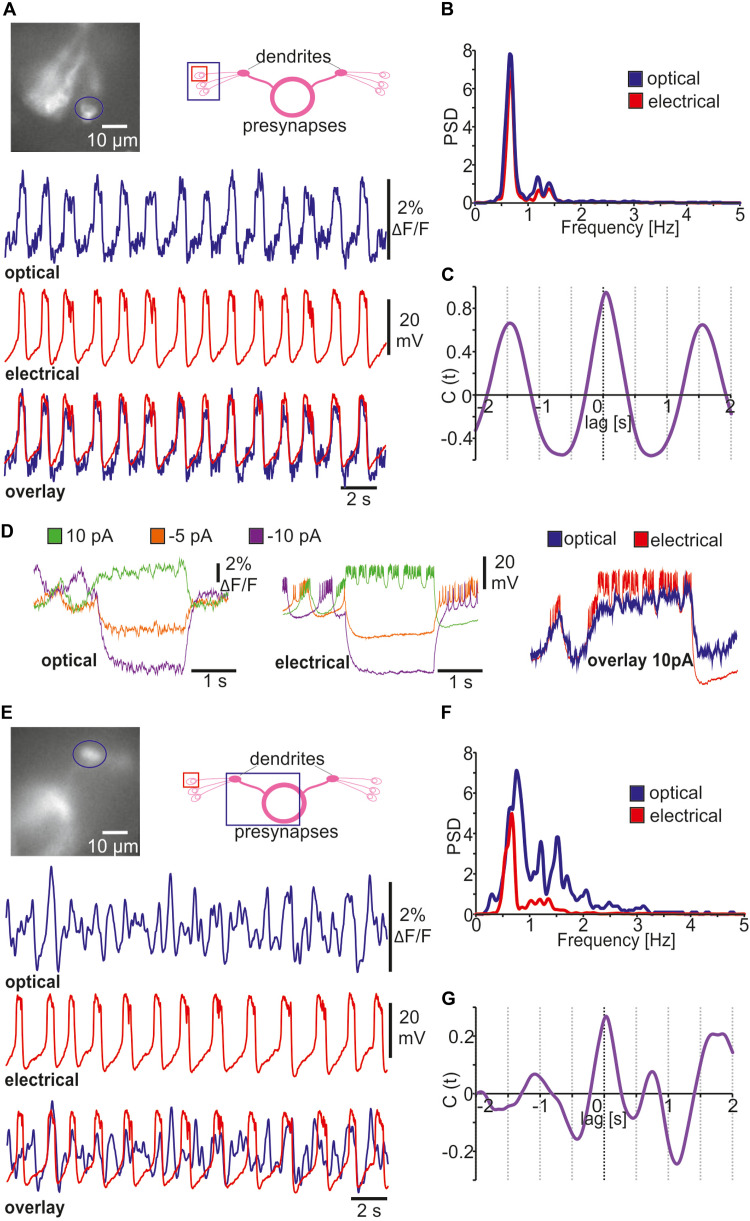
Simultaneous optical and classical electrophysiology. **(A)** Simultaneously recorded *in vivo* single-cell electrical patch-clamp (interpolated at 2 kHz) and optical recordings (ArcLight) of bursting *Drosophila* R5 neuron indicated in the wide-field image. **(B)** Power spectrum analysis of membrane potential oscillations of R5 neuron shown in A. **(C)** Cross correlograms indicating oscillatory character and temporal overlap of optical and electrical traces shown in A. **(D)** Representation of optical and electrical traces during a series of current steps. **(E)** Simultaneously recorded *in vivo* single-cell electrical patch-clamp (interpolated at 2 kHz) and optical recordings (ArcLight) of *Drosophila* R5 dendritic area indicated in the wide-field image. **(F)** Power spectrum analysis showing more complex rhythms in the optical compound activity. **(G)** Amplitude cross correlation indicating partial temporal overlap between single-cell and compound activity.

Being able to compare optical measurements alongside ongoing electrical recordings is useful in many ways. To our knowledge, NOSA is the first open-access tool to provide the option for analyzing optical and electrical traces in parallel. For example, we performed simultaneous patch-clamp and optical *in vivo* recordings from *Drosophila* R5 neurons expressing ArcLight and uploaded recordings (abf files/axon binary file format) into NOSA (Supplementary Figure S3). As electrical and optical traces may be recorded with different systems, temporal delays between the systems could falsify a direct comparison. We therefore implemented an offset function into NOSA, which allows shifting one trace in relation to the other (Supplementary Figure S3). Comparing optical and electrical traces demonstrates that ArcLight faithfully represents changes in membrane potential (Figures 6A–C) and that a change in relative fluorescence of 2% approximates a change of 23 mV in membrane potential (Figures 6A–C). However, the relation between changes in fluorescence and absolute membrane potential will highly depend on the expression strength and must therefore be determined for each cell type. To simplify a direct comparison NOSA can adjust the sampling rate via interpolation of data points. In this example we reduced the sampling rate of the electrical trace from 10 kHz to 2 kHz smoothing out the spikes on top of the bursts which in this case are not represented in the optical trace (Figure 6A). In order to compare different current or voltage steps, several optical and electrical traces can be uploaded into NOSA and the responses can be directly visualized next to each other (Figure 6D). Using the burst detection feature the response kinetics of de- and hyperpolarization events can be quantified to perform a regression analysis and thus learn more about the relation between optical and electrical responses (compare Figure 4H).

Another important application for simultaneous patch clamp and voltage imaging is to investigate single-cell contributions to population dynamics reflected in compound recordings of neural activity. Here, we use NOSA to compare simultaneously recorded *in vivo* single-cell electrical patch-clamp of a single *Drosophila* R5 neuron expressing ArcLight with the optical compound activity of the dorsal bulb, which is comprised of the dendrites of 10-12 R5 neurons (Figure 6E). To clearly visualize compound activity, we used the Savitzky Golay algorithm to remove all noise. Power spectrum analysis shows that the peak frequency of the recorded R5 neuron is also represented in the dendritic compound signal (Figure 6F). However, the power spectrum of the compound signal is much more complex due to the fact that the electrical patterns of several R5 neurons contribute to the compound signal. Correlation analysis suggests that some depolarized states of the single R5 neuron overlap with depolarized states in the compound signal (Figure 6G, compare Figure 6E).

### Cross Correlation for Analyzing Event-Based Temporal Relations

**FIGURE 7:**
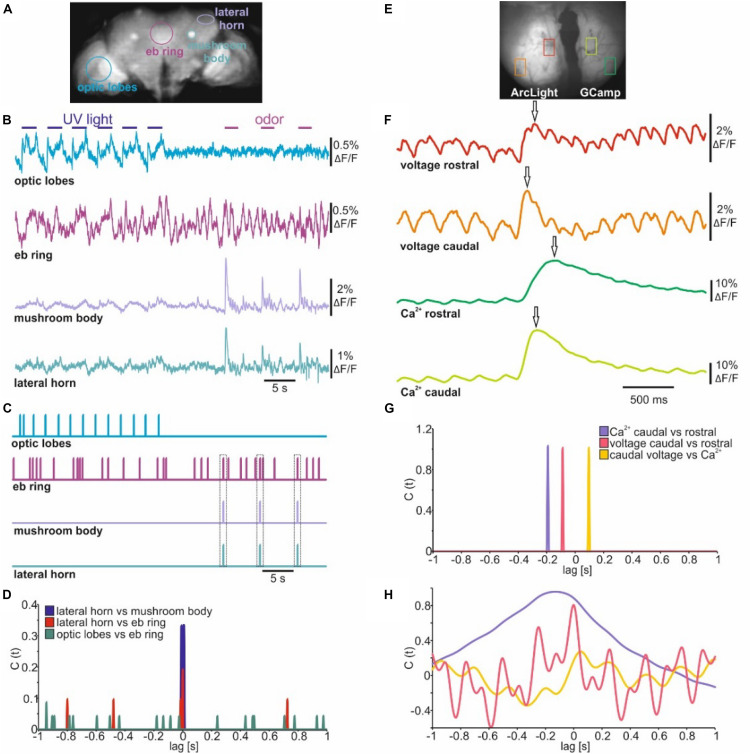
Event cross correlation identifies temporal relations in sensory evoked population dynamics. **(A)** Wide-field image of a mid-section during whole brain voltage imaging (ArcLight) in *Drosophila*. **(B)** Electrical population activity of different neuropils during optical and olfactory stimulation. **(C)** Spike detection indicating detected events of electrical activity shown in B. **(D)** Spike cross correlation indicating temporal shifts between events shown in C. Dashed squares indicate coincidence between olfactory evoked activity and specific events detected in the central complex. **(E)** Wide-field image of the olfactory bulb of C57BL/6 mice injected with ArcLight and GCamp6f in either hemisphere. **(F)** Olfactory evoked population activity of selected caudal and rostral glomeruli. Arrows indicate detected maxima. **(G)** Spike cross correlation indicating temporal shifts between maxima indicated in **(F)**. **(H)** Amplitude cross correlation indicating oscillatory character and temporal relations of population dynamics shown in **(F)**.

When imaging electrical activity in larger brain areas or even in whole brains NOSA can easily analyze the temporal relations of specific events occurring between different neuronal populations. To demonstrate this, we analyzed *in vivo* whole brain voltage imaging recordings in *Drosophila* which panneuronally express ArcLight (Aimon et al., 2019; Figure 7A). Stimulation with UV light induced electrical activity in the optic lobes while olfactory stimulation induced activity around the area of the lateral horns and peduncles of the mushroom bodies (Figure 7B), which are both higher olfactory integration centers of the *Drosophila* brain (Heisenberg, 2003; Frechter et al., 2019). Interestingly, the central complex, which processes various sensory modalities (Green et al., 2017; Sun et al., 2017) and is important for basic locomotion (Strauss and Heisenberg, 1993; Strauss, 2002), shows spontaneous electrical activity. However, it is not clear whether some of the spontaneous activity originates from the visual or olfactory stimulation (Figure 7B). Rather than detecting actual spikes, NOSA’s spike detection can be used to determine the temporal relation between detected events. Events can be detected using a linear or dynamic threshold (Figure 1F). Moreover, the threshold can be set either manually or based on the standard deviation of the whole recording. The detected events are used to generate a time-dependent event marker (Figure 7C, compare Supplementary Figure S4). The spike cross correlation function identifies the temporal relation between detected events, demonstrating that olfactory responses in the mushroom bodies and lateral horns are temporally aligned (Figure 7D). Moreover, the set event markers and cross correlation analysis reveals that olfactory stimulation generates transient activity in the central complex, while visual stimulation does not lead to a detectable response (Figure 7D).

To analyze population dynamics in a mammalian brain we used a recording in which one hemisphere of the olfactory bulb of C57BL/6 mice expresses ArcLight while the other expresses GCaMP6f (Storace et al., 2015; Figure 7E). The dorsal bulb exhibits a distinct temporal organization whereby glomeruli in the caudal bulb tend to be more coupled to respiration than glomeruli in the rostral olfactory bulb (Spors et al., 2006; Wachowiak et al., 2013). To demonstrate how quickly temporal relations can be established with NOSA we used the maxima of the olfactory responses in rostral and caudal glomeruli (Figure 7F) to generate an event-based cross correlogram (Figure 7G). This shows that electrical activity requires about 84 ms to travel from the caudal side of the olfactory bulb to the rostral side (Figure 7G). In comparison, intracellular Ca^2+^ requires about 188 ms. Moreover, at the caudal glomeruli the delay between maxima of electrical activity and intracellular Ca^2+^ is 100 ms (Figure 7G). In contrast to this event-based cross correlation, the amplitude cross correlation (Figure 7H) considers the whole recording and is thus influenced by the rhythmic changes in voltage which are a result of the mice’s breathing pattern (Storace et al., 2015).

## Discussion

Here, we report a novel open-source tool box, designed specifically for the analysis and interpretation of multicellular optical electrophysiology. Moreover, NOSA is the first open-access software that allows to combine the analysis of simultaneously recorded optical and electrical data. While there is sophisticated software for processing imaging recordings (Romano et al., 2017; Giovannucci et al., 2019), NOSA is an entirely open access stand-alone software that requires no installation and comes with an intuitive user-interface that allows to precisely control and comprehend each analytical step. In this manuscript we demonstrate the challenges of performing optical electrophysiology and provide hands-on solutions to extract and analyze electrical patterns from recordings with low signal-to-noise ratio (SNR).

We demonstrate how high recording speeds necessary to resolve single action potentials drastically reduce the SNR (Figures 3, 4). We therefore implemented baseline fitting and filtering algorithms, which can efficiently extract single action potentials and bursts from optical recordings (Figures 1–4). During voltage imaging, the issue of a low SNR is aggravated by light scattering and movement artifacts for which we implemented background subtraction and movement correction algorithms (Figure 5). We combine these basic but essential features for processing imaging data with sophisticated analysis tools for identifying electrical characteristics (Figure 4) and functional interactions (Figures 2, 6). NOSA can also be used to investigate the temporal relation of sensory-evoked population dynamics, as we demonstrate in whole brain recordings in *Drosophila* (Figures 7A–D) and in the olfactory bulb in mice (Figures 7E–H). We here show that NOSA can be used to quickly identify temporal relations between the activity patterns of single cells and neuronal populations, which is crucial for investigating under which conditions neural networks interact with each other.

The properties and limitations of GEVIs affect the optical representation of neuronal activity. For example, the improved kinetics of the red-shifted GEVI Varnam increases the likelihood of resolving single spikes while ArcLight generally yields higher SNR (Figure 3). NOSA can be used to quickly determine the properties of GEVIs. Using NOSA, we verify that the GEVI Ace2N has faster kinetics than ArcLight (Gong et al., 2015), especially with respect to the repolarization phase of an action potential (Figure 4B). However, the temporal representation of bursts is similar in both GEVIs (Figure 4G). In fact, during bursts ArcLight yields higher relative changes in fluorescence (Figure 4G). Performing a regression analysis on burst parameters automatically quantified by NOSA revealed that Ace2N, but not ArcLight responses, showed a correlation between peak amplitudes and burst duration (Figure 4H). Other important limitations of GEVIs concern the relatively slow diffusion in lipid membranes which reduces the reposition speed and thus limits the duration of imaging sessions (St-Pierre et al., 2014; Fujiwara et al., 2016). However, the bleaching kinetics strongly depend on imaging conditions and also differ between GEVIs. With specific knowledge about the properties and limitations, the adequate GEVI can be chosen for a specific experiment. A comprehensive characterization of the properties of different GEVIs are reported elsewhere (Bando et al., 2019).

NOSA’s event detection can be used to extract neuron-specific firing characteristics (Figure 4), enabling the fast identification of specific types of neurons within a population of seemingly homogeneous neurons. This knowledge could then be used to electrically stimulate neurons with specific attributes and analyze their connectivity to other neurons (Antic et al., 2016). Such sophisticated experiments would benefit from another feature, which is provided by NOSA: the simultaneous analysis of optical and electrical data (Figure 6). Moreover, we show how this feature can be used to analyze single-cell contributions to population dynamics (Figure 6E). This is especially important when trying to understand how a multitude of neurons orchestrate their electrical activities to generate population dynamics, e.g., during sleep (Buzsaki and Draguhn, 2004).

Since NOSA is implemented in Python and available on Github^1^, NOSA can easily be shared, extended to other operating systems and updated with new functions and tools. Functions that would further improve the usability of NOSA include options for hand-drawn ROIs and reliable automatic ROI detection. Implementing sophisticated artificial intelligence could provide internal performance indices (“figures of merit”) that could help the user choose the right parameters for each feature. Based on GEVI-specific kinetics, deconvolution algorithms could be used to improve the optical representation of electrical activity. For testing the functionality and reliability of such sophisticated algorithms, simulated neuronal data could be used. Simulated data has the advantage that the user can directly test whether the chosen feature and parameters can achieve the desired outcome. Moreover, an automatic regression analysis using identified response parameters would further facilitate the identification of GEVI-specific kinetics.

GEVIs are currently improving at a rapid pace, developing toward stronger fluorescence and improved kinetics. However, currently the diverse properties of GEVIs and a missing analytical pipeline represent motivational bottlenecks preventing experimental implementation and widespread use of GEVIs. With NOSA we provide an analytical toolbox that will greatly facilitate the use of GEVIs in studying multicellular electrical patterns, inexorably improving our understanding of functional interactions within neural networks.

## Data Availability Statement

The executable NOSA file, the master code and a manual containing additional information are provided under the following GitHub repository: https://github.com/DavideR2020/NOSA. To obtain example files please send an email to the lead contact.

## Ethics Statement

The animal studies were reviewed and approved by the State Office of Health and Social Affairs Berlin and the Yale Institutional Animal Case and Use Committee.

## Author Contributions

SO and FA designed and created NOSA. AE, SA, RK, DS, and DR performed the experiments. SS and JG provided resources and conceptualization. DR provided conceptualization and wrote the manuscript. All authors contributed to the article and approved the submitted version.

## Conflict of Interest

The authors declare that the research was conducted in the absence of any commercial or financial relationships that could be construed as a potential conflict of interest.

## Footnotes

1 https://github.com/DavideR2020/NOSA
