# Supplementary material for: NOSA, an Analytical Toolbox for Multicellular Optical Electrophysiology
Source: Front Neurosci. 2020 Jul 14;14:712. doi: 10.3389/fnins.2020.00712 (PMC7381214; doi:10.3389/fnins.2020.00712)
Supplement: Supplementary file 1 [file Data_Sheet_1.PDF]

Supplementary Figures

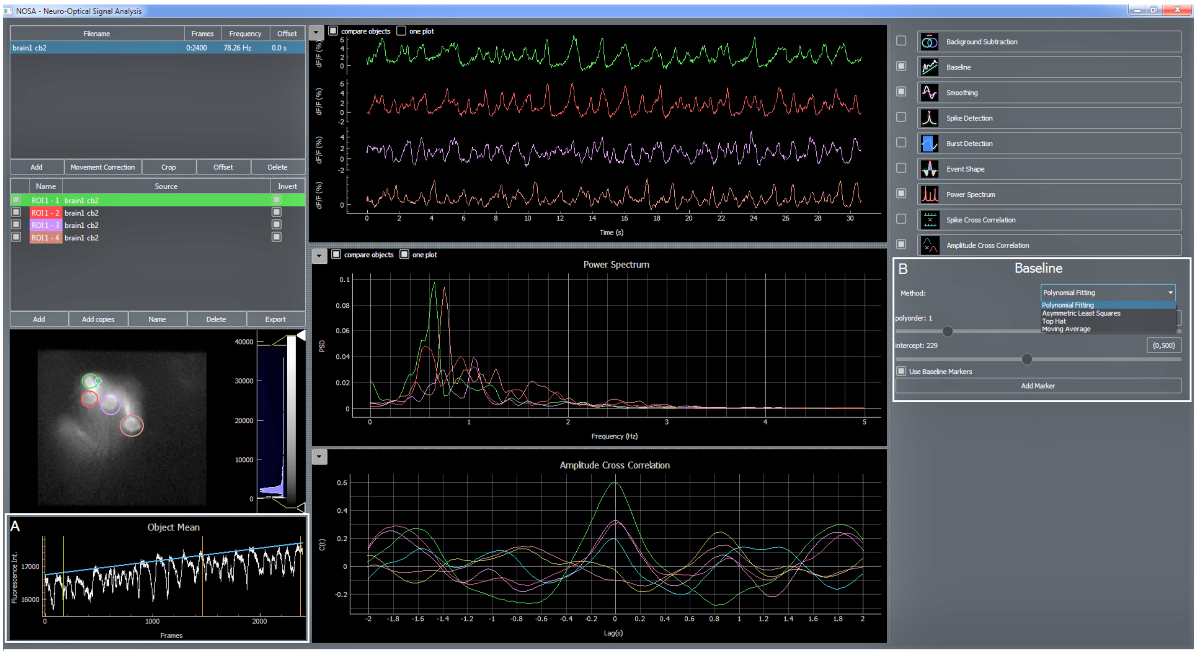

Supplementary figure 1: NOSA baseline correction. Related to Figure 2.

A, Polynomial fitting (blue curve) is used to compensate for baseline drifts and calculate the relative change in fluorescence. Additional baseline markers (orange) can be used to guide the fitting curve.

B, NOSA provides four different algorithms for baseline fitting. For each algorithm, the baseline can be adjusted by changing the shape (polyorder) and position (intercept) of the fitting curve.

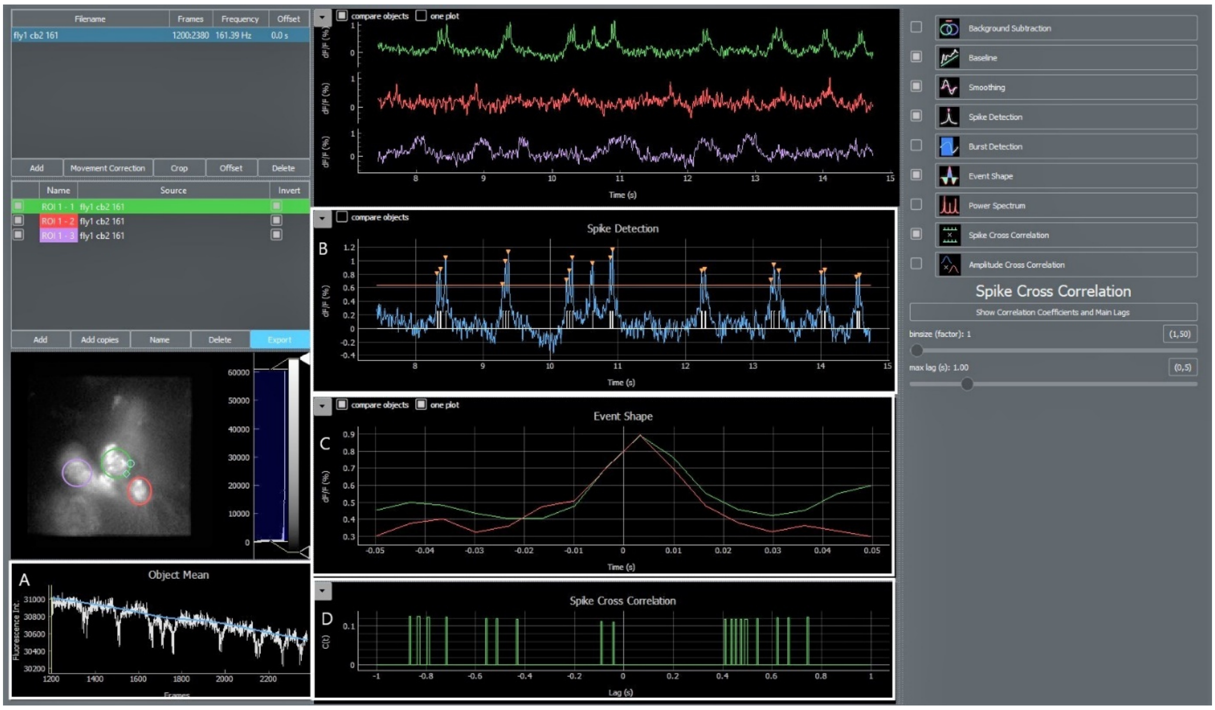

Supplementary figure 2: NOSA spike detection and cross correlation. Related to Figure 2.

A, Shift in baseline fluorescence is corrected via the asymmetric least square algorithm (blue fitting curve).

B, Spike detection using a linear threshold approach. Orange arrows indicate detected spikes.

C, Event shape can be used to generate the average shape of spikes detected in B.

D, NOSA spike cross correlation can be used to analyze the temporal relation between spikes detected in cell 1 (green) and cell 2 (red).

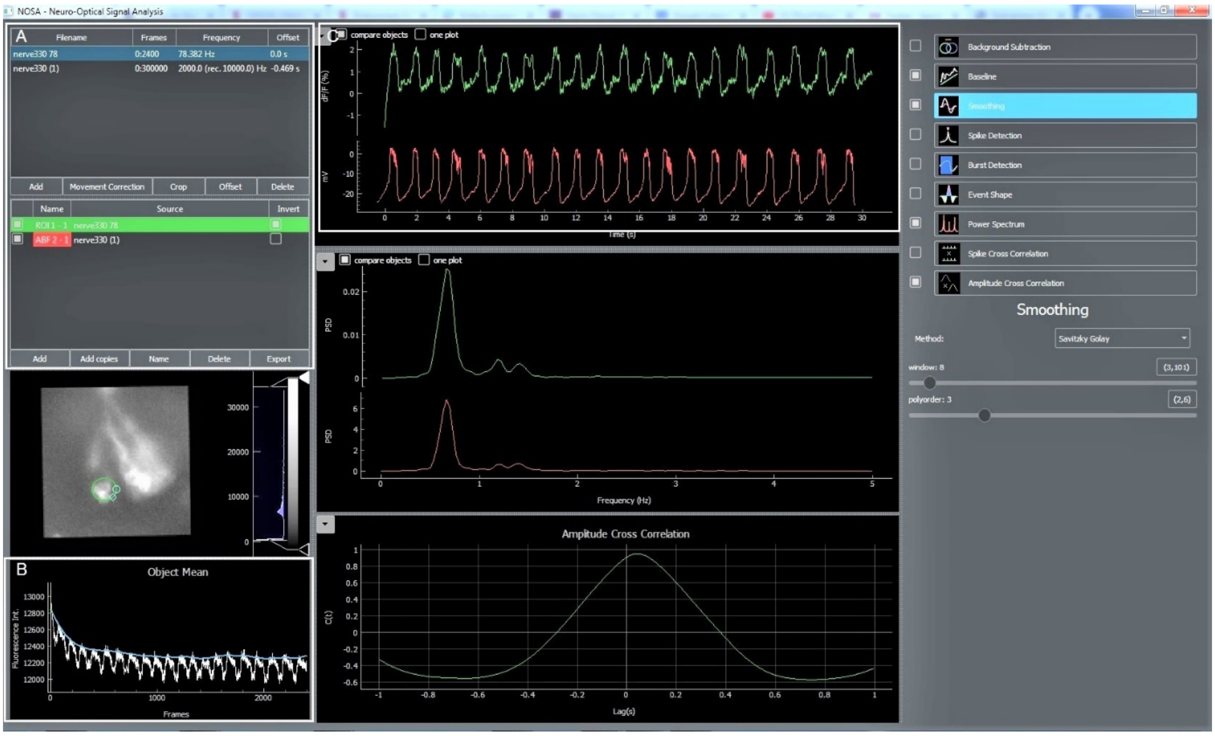

Supplementary figure 3: Simultaneous analysis of optical and electrical recordings. Related to Figure 5.

A, Optical (tiff files) and electrical (abf files) recordings can both be imported into NOSA. Sampling rates and recording speeds can be adjusted via various interpolation algorithms. The sampling rate of the electrical recording (10 kHz) was adjusted to 2 kHz. Delays between the electrical and optical systems can be corrected by an offset function (see C).

B, Optical recording was corrected for bleaching via the asymmetric least square algorithm.

C, The offset function in NOSA (see A) can adjust for temporal delays between the electrical and optical recordings. In this case, the precise delay was 469 ms.

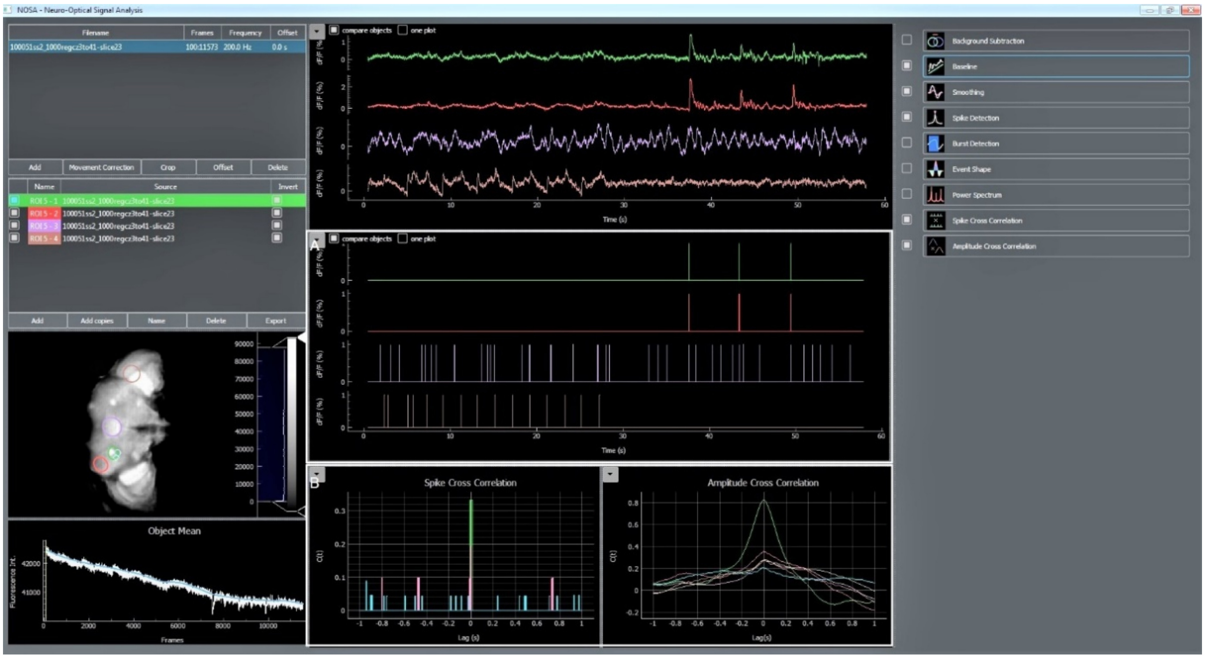

Supplementary figure 4: Combining event detection and cross correlation to identify temporal relations in population dynamics. Related to Figure 6.

A, Spike detection can be used to detect specific events, generating a time-dependent event marker.

B, Spike cross correlation calculates the temporal relation between detected events shown in A. In comparison, the amplitude cross correlation calculates temporal relations based on the relative change in fluorescence.
